# Strategies for the treatment of femoral fractures in severely injured patients: trends in over two decades from the TraumaRegister DGU^®^

**DOI:** 10.1007/s00068-020-01599-4

**Published:** 2021-02-15

**Authors:** Felix M. Bläsius, Markus Laubach, Hagen Andruszkow, Philipp Lichte, Hans-Christoph Pape, Rolf Lefering, Klemens Horst, Frank Hildebrand, Trauma Register DGU®

**Affiliations:** 1 Department of Trauma and Reconstructive Surgery, RWTH Aachen University Hospital, Aachen, Germany; 2 Centre for Regenerative Medicine, Institute of Health and Biomedical Innovation, Queensland University of Technology, Brisbane, Australia; 3 Department of Trauma, Universitaetsspital, University of Zurich, Zurich, Switzerland; 4 Faculty of Health, Institute for Research in Operative Medicine (IFOM), Witten/Herdecke University, Cologne, Germany

**Keywords:** Damage control orthopaedics, DCO, Trauma, External fixation, Early total care, ETC

## Abstract

**Purpose:**

Treatment strategies for femoral fracture stabilisation are well known to have a significant impact on the patient’s outcome. Therefore, the optimal choices for both the type of initial fracture stabilisation (external fixation/EF, early total care/ETC, conservative treatment/TC) and the best time point for conversion from temporary to definitive fixation are challenging factors.

**Patients:**

Patients aged ≥ 16 years with moderate and severe trauma documented in the TraumaRegister DGU^®^ between 2002 and 2018 were retrospectively analysed. Demographics, ISS, surgical treatment strategy (ETC vs. EF vs. TC), time for conversion to definitive care, complication (MOF, sepsis) and survival rates were analysed.

**Results:**

In total, 13,091 trauma patients were included. EF patients more often sustained high-energy trauma (car: 43.1 vs. 29.5%, *p* < 0.001), were younger (40.6 vs. 48.1 years, *p* < 0.001), were more severely injured (ISS 25.4 vs. 19.1 pts., *p* < 0.001), and had higher sepsis (11.8 vs. 5.4%, *p* < 0.001) and MOF rates (33.1 vs. 16.0%, *p* < 0.001) compared to ETC patients. A shift from ETC to EF was observed. The time until conversion decreased for femoral fractures from 9 to 8 days within the observation period. Sepsis incidences decreased in EF (20.3 to 12.3%, *p* < 0.001) and ETC (9.1–4.8%, *p* < 0.001) patients.

**Conclusions:**

Our results show the changes in the surgical treatment of severely injured patients with femur fractures over a period of almost two decades caused by the introduction of modern surgical strategies (e.g., Safe Definitive Surgery). It remains unclear which subgroups of trauma patients benefit most from these strategies.

## Introduction

The surgical treatment for severely injured patients has undergone extensive development over the past 20 years. Until the introduction of the damage control orthopaedics concept (DCO) by Scalea et al. in 2000, the early definitive care for these patients has been performed for over four decades. However, it has been observed that early definitive care leads to increased mortality rates in some subgroups of severely injured patients (e.g., unstable patients or patients with thoracic trauma). Therefore, as a bridging strategy, Scalea et al. published an early fixation of femur fractures with external fixators (EF) to avoid the burden of an early total care (ETC), thereby reducing complications, blood loss and mortality rates after major trauma [1].

Evidently, the early identification of patients at risk (borderline) is crucial and the dynamics of the posttraumatic changes require close reassessments. For the first assessment in the ER, several scoring systems have been described between 2005 and 2014. A recent comparison of the four most common scoring systems has revealed that the Polytrauma Grading Score (PTGS) appears to be the most accurate [2]. However, the study by Halvachizadeh et al. also showed that the combination of different scoring systems and pathways is more accurate in evaluating a patient’s situation than using a single scoring system. In a comparison of the four scoring systems, the PTGS proved to be the most reliable predictor of complications, while the lactate concentration showed a higher predictive value with regard to 72-h mortality. However, lactate concentrations are only of limited use in the presence of a liver injury, since this injury has a direct influence on lactate clearance and lactate accumulation can, therefore, occur [3].

Another aspect is the further development of existing resuscitation protocols towards modern ones using balanced volume therapies that prevent fluid overload and, thus, improve outcome. [4]. Moreover, point of care strategies have been included in the management of major trauma and distinct mass transfusion protocols in multiple injured patients with severe haemorrhage have been implemented [5].

All of these changes may have affected the indications for temporary external fixation versus ETC of femoral fractures and could have continuously improved the outcome of severely injured patients.

Therefore, we undertook a retrospective database analysis in a nationwide trauma registry to investigate the application frequency of different strategies (ETC, EF and conservative) for the treatment of femoral fractures in severely injured patients over the last two decades. Furthermore, we aimed to identify the factors that might influence decision making in choosing one of the aforementioned therapeutic options.

## Materials and methods

The TraumaRegister DGU^®^ (TR-DGU) of the German Trauma Society (*Deutsche Gesellschaft für Unfallchirurgie, DGU*) was founded in 1993 with the goal of creating a multi-centre database of pseudonymised and standardised documentation of severely injured patients for purposes of quality assurance and research [6]. The participating hospitals are primarily located in Germany (90%), but a rising number of hospitals in other countries have begun to contribute data as well, including Austria, Belgium, China, Finland, Luxembourg, Slovenia, Switzerland, the Netherlands and the United Arab Emirates. Currently, approximately 33,000 cases from about 650 hospitals are added to the database annually. Participation in the TR-DGU is voluntary, although hospitals associated with the TraumaNetzwerk DGU^®^ are obligated to enter at least one basic dataset for quality assurance purposes.

Data were collected prospectively over four consecutive time phases, from the site of injury until discharge from the hospital, as follows: (A) pre-hospital phase, (B) emergency room and initial surgery, (C) intensive care unit, and (D) discharge. Documentation included detailed information on demographics, injury pattern, comorbidities, pre- and in-hospital management, course of care, while in the intensive care unit (ICU), relevant laboratory findings (including data on transfusion) and clinical outcome for each individual. Inclusion criteria were admission to the hospital via the ER with subsequent ICU/ICM care or entrance to the hospital with vital signs and death prior to admission to the ICU.

The infrastructure for documentation and data management is provided by the Academy for Trauma Surgery (*Akademie der Unfallchirurgie GmbH*), a company affiliated with the German Trauma Society. Scientific leadership is provided by the Committee on Emergency Medicine, Intensive Care and Trauma Management (*Sektion NIS*) of the German Trauma Society. Participating hospitals submit pseudonymised data into a central database via a web-based application. Scientific data analysis is approved according to a peer review procedure laid down in the publication guideline of TR-DGU.

The present study is in line with the published guidelines of the TR-DGU and is registered under TR-DGU Project ID 2018-002.

### Inclusion and exclusion criteria

**Fig. 1:**
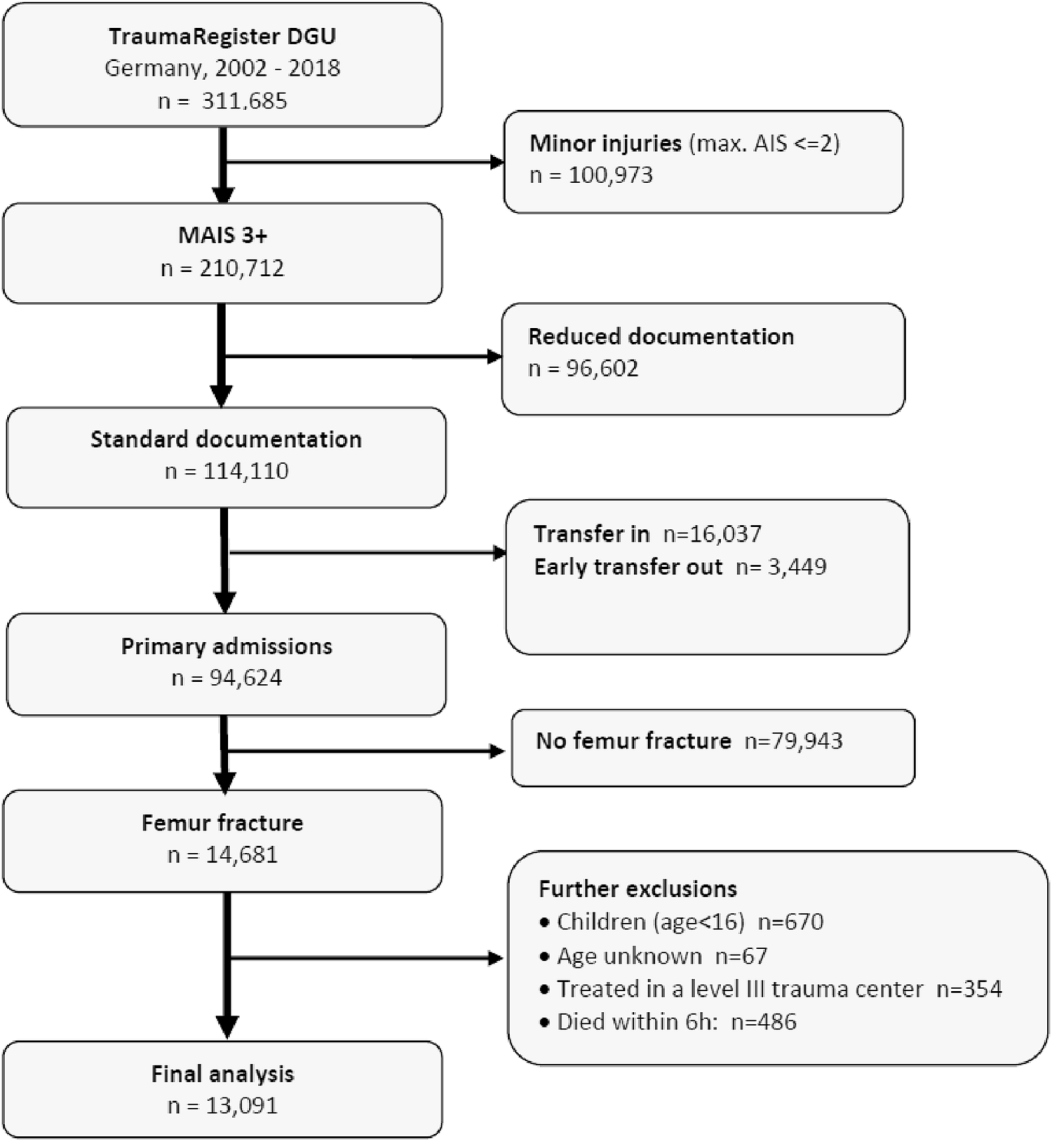
Flow diagram of the inclusion and exclusion process

The patients were identified through a retrospective record review of the TR-DGU. All trauma patients aged ≥ 16 years and treated in a German trauma centre with at least a femur fracture and a Maximum AIS (MAIS) ≥ 3 pts. between January 2002 and December 2018 were included. The resulting inclusion criterion of an Injury Severity Score (ISS) ≥ 9 pts. has been used previously to include severely injured patients [7]. Survival ≥ 6 h after admission was required (Fig. 1).

Patients who were secondarily transferred to the reporting hospital or had incomplete core datasets (e.g., lack of information about the type of surgery) were excluded.

### Definitions

The treatment concepts for femoral fractures were classified as conservative (TC) when no operative treatment was performed. This patient group includes both patients who have received immobilization in the form of a cast or brace and patients who have not received immobilization. The patients were classified as EF when the femoral fracture was treated by external fixators within the DCO concept. The definitive treatment of femoral fractures was classified as ETC. The variable “multiple fractures” comprised bilateral and multi-level femoral fractures. Injuries were coded according to the Abbreviated Injury Scale (AIS, Version 2005/2008, Association for the Advancement of Automotive Medicine, Barrington, IL). The severity of injuries was recorded according to the AIS as 1 (minor), 2 (moderate), 3 (severe, not life threatening), 4 (serious, life-threatening), 5 (critical, survival uncertain) and 6 (maximum, currently untreatable). The duration of mechanical ventilation (MV), length of ICU stay (ICU LOS) and the length of hospital stay (LOS) were recorded. The occurrence of organ failure, according to the Sequential Organ Failure Assessment Score (SOFA), was analysed [8]. Organ function was considered insufficient and defined as organ failure if the SOFA score was at least 3 points or more. MOF was defined as the simultaneous failure of at least two organs. The incidence of systemic organ impairment (sepsis and MOF) was analysed. A diagnosis of sepsis was made according to the criteria of the “Surviving Sepsis Campaign Guidelines for Management of Severe Sepsis and Septic Shock” [9]. The definition of sepsis was subjected to several revisions during the observation period. To ensure comparability, the sepsis-2 definition was applied [10]. Mortality was reported as in-hospital mortality. We used the Revised Injury Severity Score version II (RISC II) to predict the risk of death in severely injured patients who were primarily admitted to one of the reporting trauma centres [11]. RISC II scores were used to adjust the observed mortality rates by calculating the ratio of observed vs. expected mortality rate (Standardized Mortality Ratio [SMR]).

### Statistics

Categorical variables are presented as percentages only if the underlying total is obvious. Metric data are presented as mean and median with standard deviation (SD). Differences in categorical and metric variables were evaluated with the chi-squared test and Mann–Whitney *U* test, respectively. The changes over time were analysed on a yearly basis using linear regression analysis or with the chi-squared test where appropriate. Due to the large sample size, even minor differences could become statistically significant. Therefore, the interpretation of results should focus on the clinical relevance of a difference rather than on statistical significance. All statistical analyses were performed using SPSS statistical software (SPSS 25.0; IBM Inc., Armonk, NY, USA).

## Results

### Patient data

In total, 13,091 patients met the inclusion criteria during the 17-year observation period. The majority of the patients were male (72.4%) and the mean age was 44.4 years (SD 21.0). In our cohort, the patients predominantly suffered from blunt trauma (97.2%). The injury mechanisms and baseline characteristics are shown in Table 1.

**Table 1:** Demographics, trauma mechanisms, injury pattern and outcome. Continuous values are presented as mean / median (standard deviation)

	TC	ETC	EF	Total
*n*	1601	5249	6241	13,091
Demographics				
Age, mean (SD)	47.0 (21.7)	48.1 (22.3)	40.6 (18.9)	44.4 (21.0)
Male (%)	70.5	70.6	74.3	72.4
Penetrating Injury mechanisms (%)	2.9	2.1	3.4	2.8
Car (%)	30.3	29.5	43.1	36.1
Motorcycle (%)	23.3	20.2	27.6	24.1
Bicycle (%)	5.3	4.6	2.9	3.9
Pedestrian (%)	7.5	5.2	5.0	5.4
High fall > 3 m (%)	17.3	19.4	14.0	16.6
Low fall < 3 m (%)	10.7	14.9	2.8	8.6
Outcome				
ICU stay (%)	83.8	91.9	96.7	93.2
ICU LOS, mean/median (SD), day	8.2/3 (13.3)	7.6/3 (11.5)	13.2/8 (15.7)	10.3/5 (14.1)
Ventilation time, mean/median (SD), day	4.2/0 (9.3)	3.8/1 (9.0)	7.4/2 (12.4)	5.6/1 (10.9)
Ventilator-free days, mean (SD)	22.0 (11.9)	25.7 (8.4)	21.8 (10.6)	23.4 (10.1)
LOS, mean/median (SD)	24.8/18 (25.2)	23.7/18 (20.4)	32.9/26 (25.9)	28.3/22 (24.2)
MOF (%)	30.2	16.0	33.1	25.9
Sepsis (%)	7.4	5.4	11.8	8.8
Mortality (%)	24.5	5.0	8.7	9.1
RISC II prognosis (%)	18.8	5.9	10.4	9.6
SMR [95% CI]	1.30 [1.19–1.41]	0.85 [0.75–0.95]	0.83 [0.77–0.90]	
Injury characteristics				
ISS, mean (SD)	26.1 (15.4)	19.1 (10.7)	25.4 (13.4)	23.0 (13.0)
ISS ≥ 16 (%)	71.3	53.5	73.9	65.4
AIS head ≥ 3 (%)	29.4	17.0	27.5	23.5
AIS thorax ≥ 3 (%)	46.2	31.7	49.8	42.1
AIS abdomen ≥ 3 (%)	14.1	9.2	17.5	13.7
Multiple femur fractures (%)	3.7	5.1	14.9	9.6

Regarding the mode of accident, the proportion of patients involved in traffic accidents (car, motorcycle) was higher in the EF than in the ETC group (Table 1). Significant differences were also noted for the occurrence of MOF (33.1% vs. 16.0%, *p* < 0.001) and sepsis (11.8% vs. 5.4%, *p* < 0.001), with a higher prevalence in EF patients. The ETC group had significantly shorter ICU LOS and LOS, as well as a shorter duration of MV, compared to EF patients (Table 1).

### Injury severity, injury characteristics and surgical treatment

**Fig. 2:**
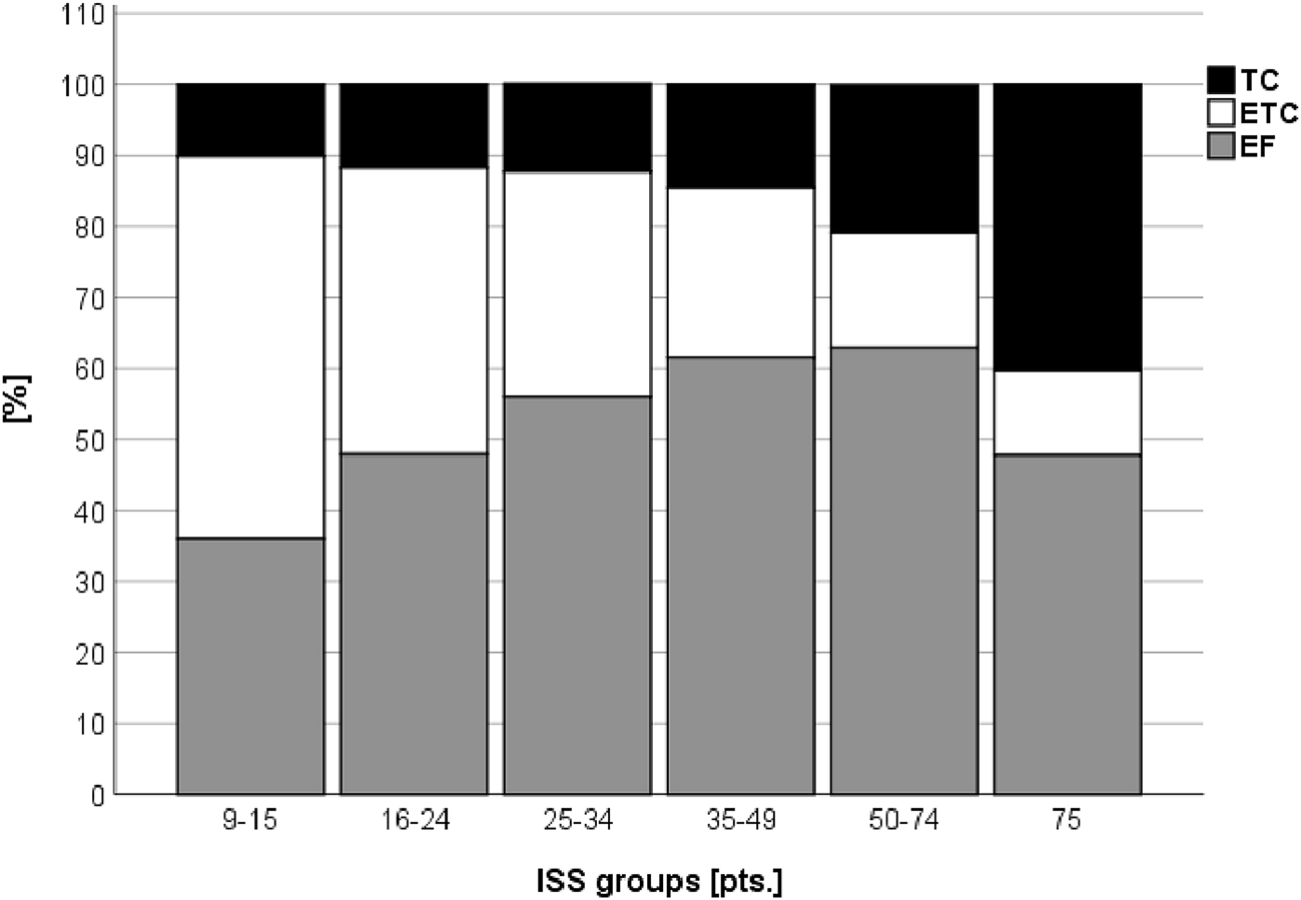
Proportion of TC, ETC or EF according to injury severity (ISS)

The mean ISS was higher in EF compared to ETC patients (ISS 25.4 pts. SD 13.4 vs. 19.1 pts. SD 10.7, p < 0.001). Moderately injured patients (ISS 9–15 pts.) predominately underwent ETC treatment (53.9% vs. 36.0%, *p* < 0.001), while higher ISS values were associated with an increased application of EF (ISS 50–74 pts.: 16.2 vs. 62.9%, *p* < 0.001) (Fig. 2). Moreover, EF patients showed a higher proportion of multiple femur fractures compared to ETC and TC patients (14.9% vs. 5.1% vs. 3.7%, *p* < 0.001) (Table 1). Additionally, a relevant thoracic trauma was more often observed in the EF group compared to the ETC group (49.8% vs. 31.7%, *p* < 0.001). Similarly, severe abdominal (17.5% vs. 9.2%, *p* < 0.001) and head injuries (27.5% vs. 17.0%, *p* < 0.001) were more frequently found in the EF group.

### Age and surgical treatment

**Fig. 3:**
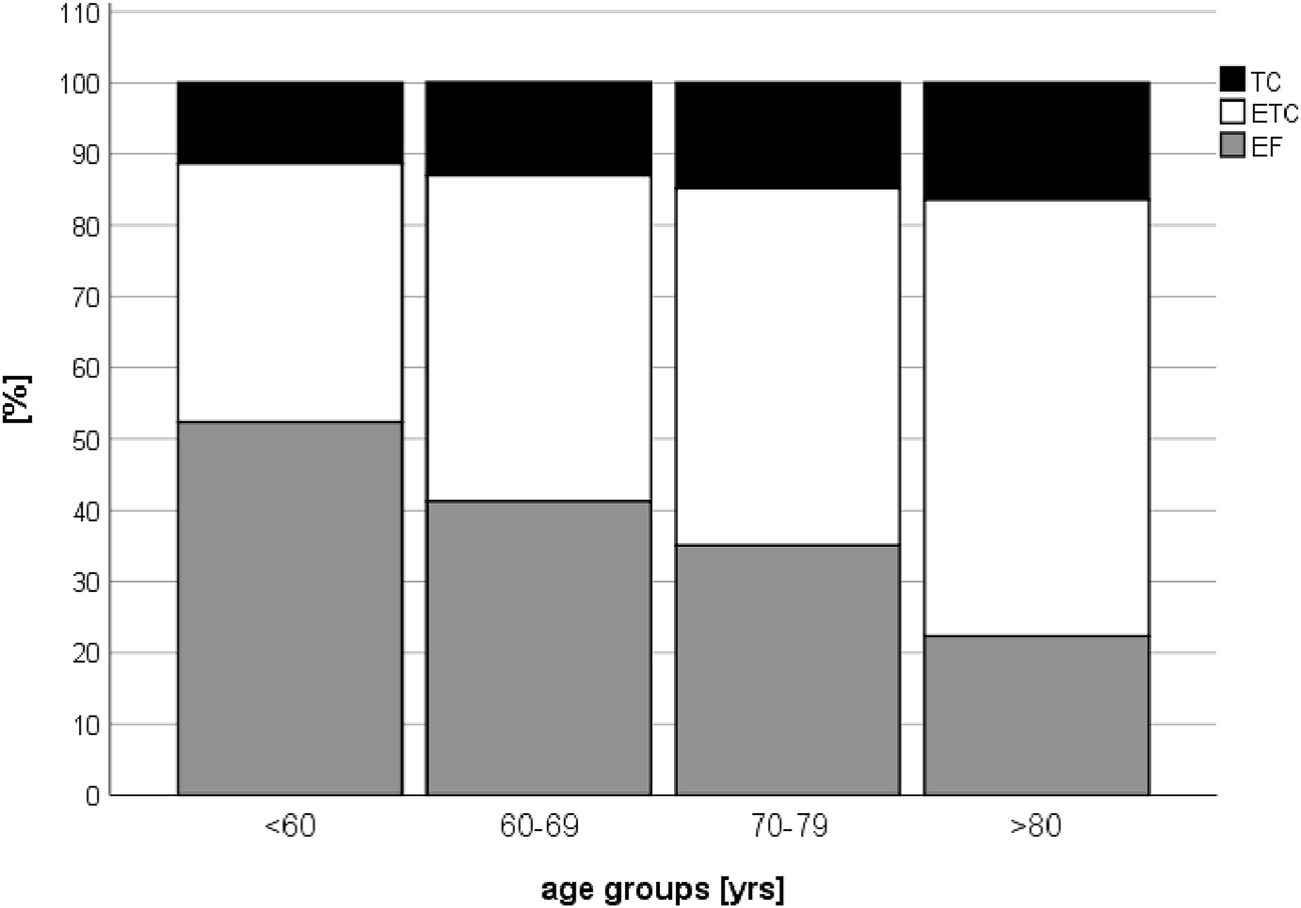
Proportion of TC, ETC or EF according to the different age groups

The EF group was composed of younger patients than the ETC group (40.6 vs. 48.1 years, *p* < 0.001). A subgroup analysis of the different age groups showed that ETC and TC were predominantly chosen with increase in age. For patients under 60 years, ETC treatment was performed in 38.4% of all cases, whereas 47.1% underwent EF treatment. For those older than 80 years, majority of the patients were treated with ETC. The proportion of patients with TC increased with the increase in age from 14.5 to 27.4% (Fig. 3). At the same time, there were no significant differences between age groups 16–59 (mean ISS 23.5 pts.), 60–69 (mean ISS 23.2 pts.) and 70–79 years (mean ISS 22.5 pts.) with regards to ISS. Patients aged ≥ 80 years showed lower ISS values (18.4 pts.) compared to the other age groups (*p* < 0.001).

### Changes within the observation period

**Fig. 4:**
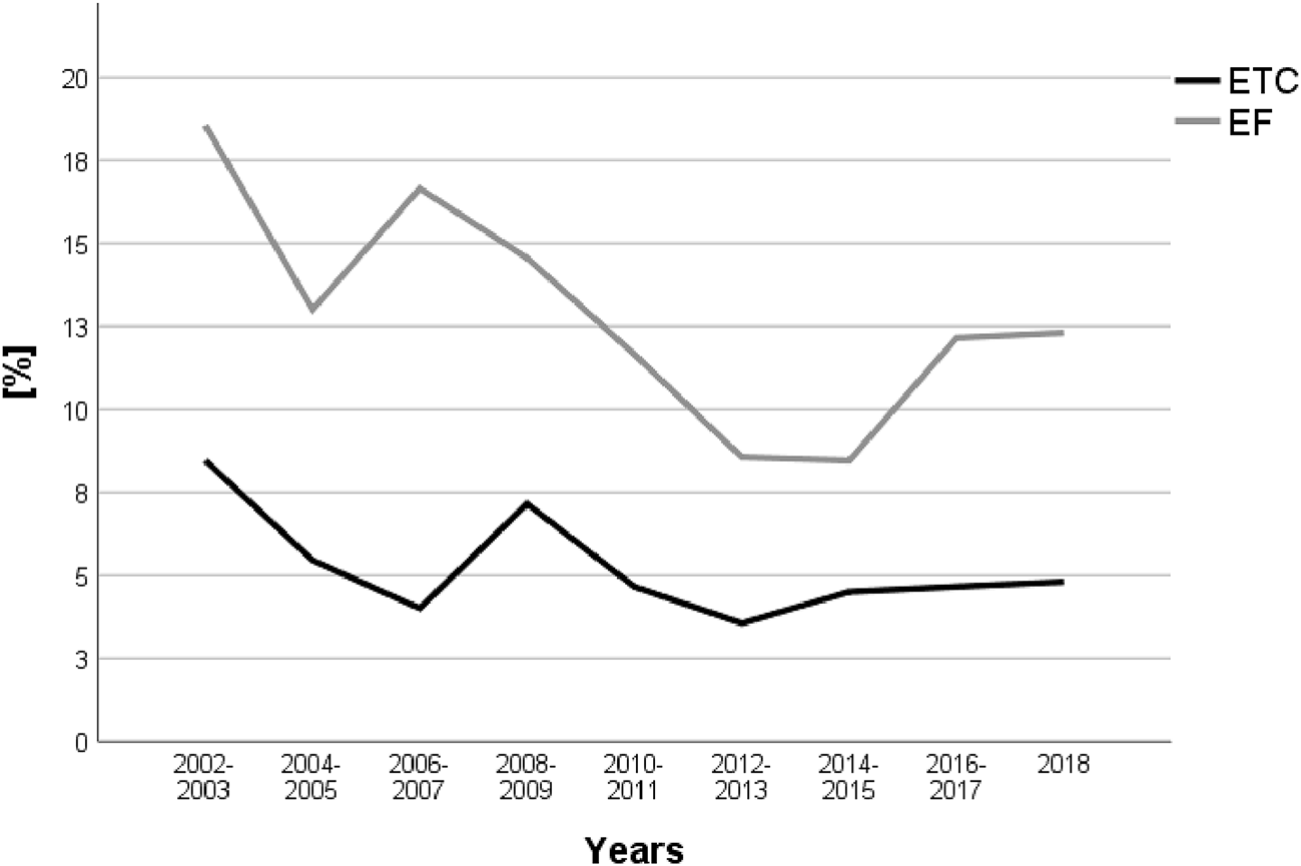
Sepsis rates

**Fig. 5:**
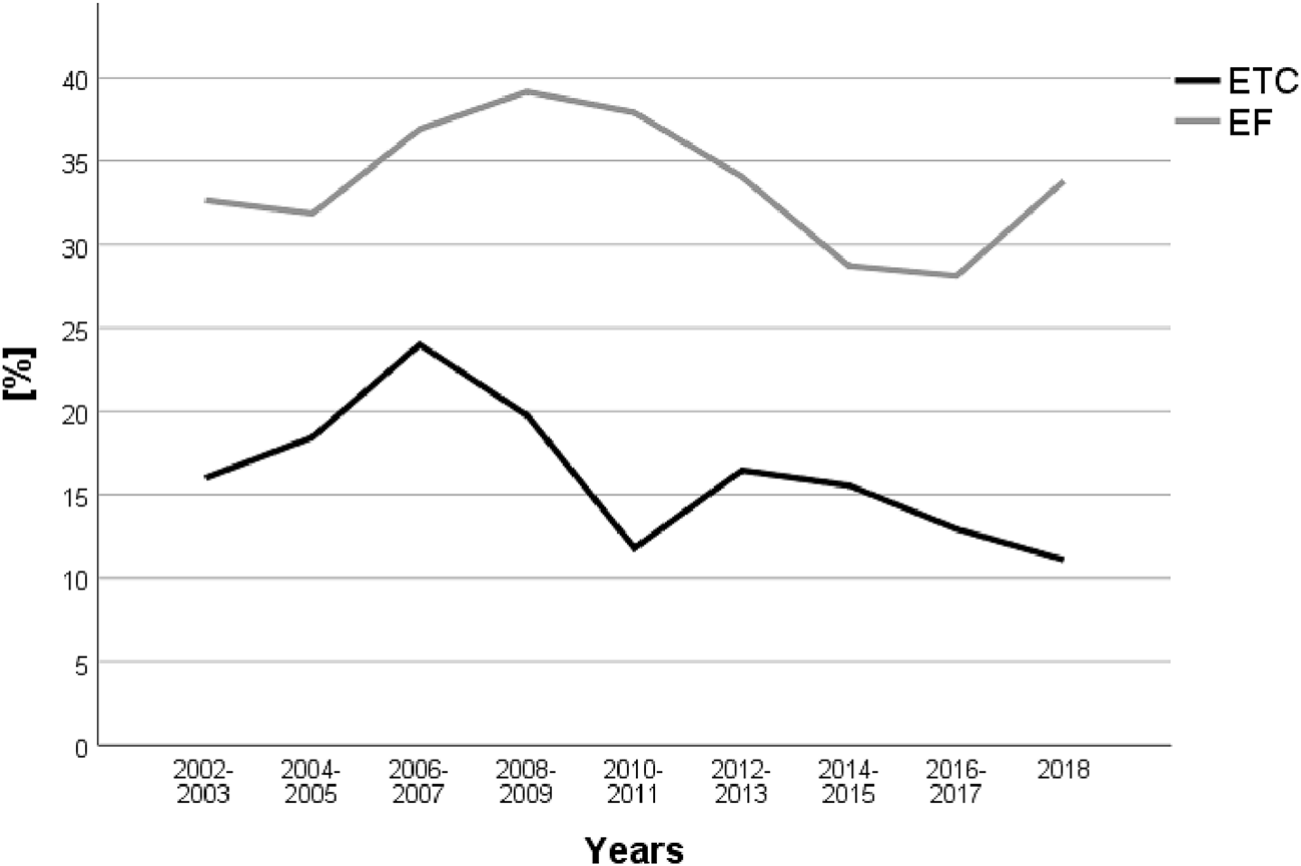
MOF rates

During the observation period, the mean age increased from 40.3 years in 2002 to 48.6 years in 2018 (*p* < 0.001). The ISS was stable at around 22 points. EF rates increased during the observation period, from 36.5% in 2002 to 51.2% in 2018 (*p* < 0.001). Accordingly, ETC rates declined from 53.2% in 2002 to 38.5% in 2018 (p < 0.001, Table 2). Sepsis rates decreased in the ETC and EF groups (Fig. 4), while MOF rates remained stable (Fig. 5). SMR remained stable during the observation period, accompanied by stable ISS values of 22–23 points and in accordance to a predicted decrease by RISC II prognosis (11.1% to 9.6% risk of death, *p* < 0.001, linear regression) (Table 2). Time until conversion to definitive treatment in the EF group decreased from 9 days in 2002 to 8 days in 2018.

**Table 2:** Changes from 2002–2018

Year	*n*	Age mean	Male (%)	ISS mean	Mortality (%)	RISC II (%)	SMR	ETC (%)	EF (%)
2002	342	40.3	71.1	22.4	13.5	11.1	1.22	53.2	36.5
2003	373	42.8	73.2	23.1	13.1	13.1	1.0	52.3	42.1
2004	379	39.6	74.4	22.2	12.4	11.6	1.07	57.5	30.9
2005	391	42.0	70.3	23.5	13.8	13.1	1.05	48.1	43.5
2006	481	40.2	71.9	24.8	12.1	13.6	0.89	49.9	39.9
2007	760	40.4	74.6	25.3	10.4	12.0	0.87	44.3	46.1
2008	696	40.4	74.1	25.9	12.6	14.0	0.9	47.4	44.0
2009	624	41.4	75.0	25.5	9.9	10.1	0.98	34.5	41.7
2010	742	42.7	70.0	24.1	6.7	8.8	0.76	30.9	42.2
2011	913	41.7	75.8	23.2	6.7	8.2	0.82	33.4	53.8
2012	1000	44.8	72.2	23.0	9.0	8.7	1.03	32.3	55.9
2013	987	45.1	72.2	22.3	7.4	8.5	0.87	34.3	51.2
2014	1034	45.1	73.8	21.7	7.6	8.4	0.9	39.5	50.8
2015	1121	47.7	72.3	21.1	7.3	7.6	0.96	40.9	50.2
2016	1094	48.0	69.7	22.5	8.5	8.7	0.98	38.8	48.8
2017	1143	48.0	68.8	21.5	7.8	7.8	1.0	41.0	48.6
2018	1011	48.6	72.6	22.2	9.5	9.4	1.01	38.5	51.2
Total	13,091	44.4	72.4	23.0	9.1	9.6	0.95	40.1	47.7

Demographics, injury severity (ISS), mortality, RISC II and ETC/EF proportion between 2002 and 2018

## Discussion

The strategy for initial fixation of femoral fractures is well known to have a significant effect on the clinical course after severe trauma. The avoidance of the burden of early definitive treatment in the DCO concept has been shown to reduce the incidence of complications in certain subgroups of severely injured patients. In this study, we focused on the application changes of these surgical strategies over an observation period of almost two decades to identify the factors that might influence decision making. The main findings obtained in our study can be summarised as follows:

1. An increasing overall injury severity, severe head, chest and abdominal trauma, as well as the presence of multi-level/bilateral femoral fractures, were associated with the application of the DCO concept.
2. Despite a comparable injury severity, a significant shift from ETC towards EF was observed over the observation period.
3. ETC was the preferred treatment for elderly patients.
4. The time until conversion from EF to definitive fracture stabilisation decreased within the observation period.
5. Mortality rates remained almost stable within the observation period. Subgroup analyses revealed that the incidence of sepsis decreased in ETC and EF patients, while the incidence of MOF remained stable in both groups.

Our results confirmed that EF is particularly used in patients with high overall injury severity and is associated with high-energy motor vehicle accidents. More specifically, severe head, chest and abdominal trauma represented indications for the DCO concept. The findings obtained in our study are in line with the results of former studies [12–14]. It is worth noting that the EF rates significantly increased at the expense of ETC treatment within the observation period, although the overall injury severity remained stable. This observation might be explained by different aspects. First, it might be assumed that the principles of DCO for femoral fracture fixation have gained more acceptance within the care of severely injured patients, resulting in its more widespread use in German trauma centres. Second, independent from injury severity and the general status of the patient, EF may have been indicated for soft tissue protection in open fractures and high-energy injuries. Especially with regard to grade III open tibia fractures, EF remains the preferred mode of stabilisation [15]. Moreover, fracture morphology is well known to have a significant influence on the choice of the treatment strategy. Both the complexity of the fracture and the presence of bilateral and/or multi-level femoral fractures might guide the therapy towards DCO. This is in line with our results that showed significantly higher rates of multiple femur fractures in the EF group (Table 1).

It might be surprising that around 12% of the patients included in our study were treated conservatively. However, the highest injury severity and the most pronounced mortality rate in this group of patients clearly indicated that femoral fractures in patients with an infaust prognoses were treated non-operatively. Moreover, could have been classified as conservatively treated patients who, due to the severity of their injuries, had not undergone the surgical therapy, because they had died before. This argument is also supported by the SMR of this patient group, which is significantly increased compared to the other groups.

Interestingly, our study also revealed age-related effects on the surgical strategy. In this context, a lower application rate of EF in older patients was observed. The choice for ETC or TC may be ascribed to different reasons. On the one hand, it is well known that the older population benefits from early mobilisation to prevent complications and to maintain mobility [16]. As ETC is associated with earlier mobilisation, this might explain the higher incidence of ETC in older patients [17]. The shortest LOS, ICU LOS and ventilator-free days showed the correlation between early mobilisation and shorter recovery times with ETC treatment. Additionally, ETC prevents patients from undertaking a second surgical procedure, which is also well known to result in a delay of posttraumatic recovery. Therefore, the preferred use of ETC might represent the general idea to quickly mobilise these patients to avoid prolonged treatment, complications and early death. On the other hand, a higher proportion of pre-existing diseases and a lower demand on posttraumatic functionality with a higher acceptance of dislocation in this age group might have resulted in more restrained indications for operative treatment, resulting in a higher proportion of TC. Furthermore, in older people presenting with a relatively high ISS, TC might have been the treatment of choice, particularly when life-threatening and infaust injuries were present. In these cases, life expectancy might have been limited due to restricted physiological reserves and an additional surgical impact was avoided to avoid worsening the patient’s situation; thus, a decision for conservative treatment was made [18, 19]. Moreover, the fracture pattern in elderly patients might be different. Proximal femur fractures may have occurred more frequently in this patient group. Stabilization of these fractures is usually performed by ETC. Stabilization by EF or conservative therapy is usually neither necessary nor practical. These age-related specifics are likely to gain increasing importance in the future due to a rising mean age of severely injured patients, which we also observed in our study. This trend among older patients can be explained by the general demographic developments, as well as the increasing mobility and activity of the ageing population in Western industrialised countries [20].

Within the DCO concept, the optimal time point for conversion from EF to definitive stabilisation is of utmost importance to avoid complications and to optimise outcome. In our study, the mean time until definitive fixation over the entire observation period was around 8 days. This timing largely varies in the literature [21, 22]. For example, Pape et al. reported a mean duration of external fixation of 4.6 days in a cohort of multiple-injury patients with ISS ≥ 18 and femoral shaft fractures [23], whereas Taeger et al. showed a much longer duration of external fixation (mean 13 days) in a cohort of 679 patients with ISS ≥ 16 [24]. These differences are likely to be at least partly caused by different study designs with diverse inclusion criteria. In this context, Taeger et al. included patients with at least two extremity fractures and a pelvic fracture, whereas Pape et al. focused on patients with bilateral femoral fractures. The results of our study showed a clear trend towards a reduced time period until definitive stabilisation after the initial EF over the observation period. It may be assumed that standard operating procedures, as well as perioperative and intensive care, have improved since the early 2000s; thus, conversion times may have been synchronised and shortened [6]. This synchronisation is associated with the introduction of a modification of the DCO concept called Safe Definitive Surgery (SDS). SDS represents a synthesis of the ETC and the DCO strategy and is characterised by short-term re-assessments of a patient’s status to dynamically adapt the time point of conversion to definitive fixation [25].

In parallel to the aforementioned increase of EF rates, we found stable SMR values over the observation period. Although the establishment of structured surgical treatment concepts has been proven to beneficially influence outcomes after severe trauma, we were not able to demonstrate a correlation between the strategy of fracture fixation and mortality due to the design of our analysis [26]. Furthermore, the reasons for the decreasing SMR in the early years (until 2008) are likely to be multifactorial. First, the implementation of regional trauma systems has been shown to contribute to decreasing mortality rates [27]. Moreover, other studies have provided evidence that improved pre-hospital (e.g., Prehospital Trauma Life Support®) and in-hospital (e.g., Advanced Trauma Life Support®) trauma care protocols help reduce mortality [28]. Improvements in intensive care (e.g., early prevention strategies for pneumonia and low tidal volume ventilation) have also contributed to the decrease of trauma-related mortality. In this context, we and others have noted a reduction in sepsis rates over the observation period [29–31]. Interestingly, the overall incidence of MOF remained stable in our cohort, which indicates that severe infections are not the main cause for the development of posttraumatic MOF. Due to the limitations of a registry analysis, it remains unclear from our data whether the strategy for fracture fixation has a significant impact on the incidence of these complications due to the avoidance of a surgery-related second hit phenomenon. One of the few attempts of a randomized controlled multicenter trial on damage control orthopaedics was registered in 2009 by Rixen et al. Unfortunately, this study had to be terminated in 2016 due to insufficient patient recruitment without statistically exploitable data [32].

However, the higher incidences of MOF and sepsis in the EF-treated patients are most likely caused by the increased overall injury severity, as well as the higher incidences of severe head, chest and abdominal trauma, which have all been identified as relevant risk factors for adverse outcome after trauma [33].

### Strengths and limitations

The number of patients included in this cohort study gives rise to important statements about this patient group. Notably, an observation time of almost two decades is striking. However, retrospective registry studies are restricted to the initially documented data, although the data quality of the TR-DGU is accepted as being high [34]. The registry used the AIS-1998 version until 2008. Since 2009, the TR-DGU has used a reduced version of the AIS-2005/08, where similar codes with the same severity level were merged but differing severity levels were preserved. The datasets included before 2009 were adapted with respect to the new codes.

## Conclusions

The strategies for surgical treatment of femoral fractures in severely injured patients has undergone a significant change over the past two decades. Over this period, the DCO concept has significantly gained importance. However, the timing until conversion to definitive treatment has shortened. This finding is reflected by the introduction of new surgical concepts (e.g., SDS) whose aim is short-term re-assessments and dynamic adaption of the time point of definitive fixation. These concepts seem inapplicable among elderly patients for whom ETC or conservative treatment is the therapy of choice. Once again, our study clearly demonstrates the need for randomised prospective studies to identify the subgroups of trauma patients that benefit most from either of the aforementioned surgical strategies.

## Data Availability

The data were obtained from the TraumaRegister DGU^®^. Data are available from the TraumaRegister DGU® for researchers who meet the criteria for access to confidential data.

## Appendix

See Figs. 1, 2, 3, 4 and 5 and Tables 1 and 2.

## Abbreviations

AIS: Abbreviated injury scale
ARDS: Acute respiratory distress syndrome
TC: Conservative treatment
DCO: Damage control orthopaedics
DGU: Deutsche gesellschaft für unfallchirurgie
EF: Temporary external fixation
ETC: Early TOTAL Care
ICU: Intensive care unit
IMC: Intermediate care
ISS: Injury severity score
MAIS: Maximum abbreviated injury scale
MOF: Multiple organ failure
MV: Mechanical ventilation
RISC II: Revised injury severity score II
SDS: Safe definitive surgery
SOFA: Sequential organ failure assessment
TR-DGU: TraumaRegister DGU®
